# Salt-tolerant alternative crops as sources of quality food to mitigate the negative impact of salinity on agricultural production

**DOI:** 10.3389/fpls.2023.1092885

**Published:** 2023-02-03

**Authors:** Isabel Egea, Yanira Estrada, Celia Faura, José M. Egea-Fernández, Maria C. Bolarin, Francisco B. Flores

**Affiliations:** ^1^ Department Of Stress Biology and Plant Pathology, Centro de Edafologia y Biologia Aplicada del Segura (CEBAS-CSIC), Universidad de Murcia, Murcia, Spain; ^2^ Department of Plant Biology, Faculty of Biology, University of Murcia, Murcia, Spain

**Keywords:** tomato landraces, amaranth, quinoa, production, adaptation, tolerance costs, quality, salinity

## Abstract

An increase of abiotic stress tolerance and nutritive value of foods is currently a priority because of climate change and rising world population. Among abiotic stresses, salt stress is one of the main problems in agriculture. Mounting urbanization and industrialization, and increasing global food demand, are pressing farmers to make use of marginal lands affected by salinity and low-quality saline water. In that situation, one of the most promising approaches is searching for new sources of genetic variation like salt-tolerant alternative crops or underexploited crops. They are generally less efficient than cultivated crops in optimal conditions due to lower yield but represent an alternative in stressful growth conditions. In this review, we summarize the advances achieved in research on underexploited species differing in their genetic nature. First, we highlight advances in research on salt tolerance of traditional varieties of tomato or landraces; varieties selected and developed by smallholder farmers for adaptation to their local environments showing specific attractive fruit quality traits. We remark advances attained in screening a collection of tomato traditional varieties gathered in Spanish Southeast, a very productive region which environment is extremely stressing. Second, we explore the opportunities of exploiting the natural variation of halophytes, in particular quinoa and amaranth. The adaptation of both species in stressful growth conditions is becoming an increasingly important issue, especially for their cultivation in arid and semiarid areas prone to be affected by salinity. Here we present a project developed in Spanish Southeast, where quinoa and amaranth varieties are being adapted for their culture under abiotic stress targeting high quality grain.

## Introduction: The salinity challenge

Climate change is having dramatic consequences on agriculture worldwide due to the multiple abiotic stresses pressing on plants including salinity. Currently, it is estimated that about 40% of the Earth’s surface is under water and salt stress and could increase up to 50% in next years. But the impact of salinity on agriculture will greatly increase in next years because cities and industries will need to use more water of good quality leaving saline waters for agriculture. Therefore, the greatest challenge for the short-term future in agriculture will be to increase crop production under salinity conditions (Munns et al., 2020).

Plant tolerance to stress is the result of different processes with contrasting above and belowground physiological responses. Although important advances have been achieved, there is still different and sometimes contradictory results on the main physiological traits coordinating salt tolerance in roots and shoots. One of the causes is that salinity experiments have been performed rather far away from realistic agronomic scenarios, by using high stress levels where the plants cannot complete their life cycle and, therefore, the selected physiological traits and genes are those involved in plant survival under salt stress (Egea et al., 2022). Tolerance to salinity varies not only with the level of stress intensity but also with the stage of plant development and the exposure time to salt stress. Thus, numerous studies have been carried out at the germination stage, or in young plants without highly vacuolated cells. On the other hand, the salt responses are frequently analyzed at short times of salt exposure, where cell homeostasis is not recovered yet and, therefore, the changes are not directly associated with the plant tolerance to long-term (Flowers et al., 2015). Another factor to take into consideration is the temporal variability of soil salinity, such as occurs in coastal areas, where the groundwater depth and soil salinity greatly vary as the result of the variation of seawater level (Liu et al., 2020a). In this picture, it is necessary to analyze the different components operating simultaneously and interacting to a greater or lesser extent in order to provide the overall salinity tolerance. These are mainly the tolerance to the osmotic stress imposed by salinity, exclusion of Na^+^ from the shoot, and tissue tolerance to accumulation of Na^+^.

To reduce the osmotic stress induced by salinity, the ability to reduce water loss through the leaves is an important salt tolerance mechanism found in salt-tolerant species. In addition to measures of stomatal conductance and transpiration, the infrared thermography has been used as a rapid and non-destructive methodology for estimating the water loss through the leaves by determinating changes of temperature. While control of stomatal conductance (g_s_) is essential to maintain a correct balance between CO_2_ assimilation and transpirational water loss under saline conditions, a substantial amount of water evaporated from the leaf surface may bypass stomata through the cuticle, and the contribution of this non-stomatal component can be as high as 28% of the total amount of water transpired through stomata (Shabala et al., 2013). But the salt tolerance has also been related to reduced stomatal density in both abaxial and adaxial surfaces, as demonstrated in the wild tomato salt-tolerant species *Solanum pennellii* (Albaladejo et al., 2017). This is a consequence of the non-controlled water loss through the pores around the stomata, so the lower stomatal density, the lower number of pores through which water is lost around the stoma (Kiani –Pouya et al., 2019). Decreases in stomatal density with increasing salinity have also been found in several highly salt tolerant halophyte species, which supports that this mechanism might represent a fundamental strategy by which a plant may optimize water retention under saline conditions (Liu et al., 2020b). Moreover, the toxic ion Na^+^ is transported from root to shoot through the transpiration stream and, therefore, Na^+^ toxicity levels may be indirectly achieved in the leaf as consequence of high leaf transpiration. Thus, the analysis of the *ars1* tomato mutant allowed the identification of the first R1-MYB type transcription factor involved in salt tolerance, as the *ARS1* gene disruption deregulated stomatal closure under salt stress, increasing leaf transpiration and thus the massive Na^+^ transport up to the leaves (Campos et al., 2016).

In last years, variation in leaf structure has also been considered important in abiotic stress tolerance (Muir et al., 2014; Rowland et al., 2020). It is coherent with the fact that the increased ability to avoid water loss under stressful conditions is associated with the size of leaf mesophyll cells. In the wild salt-tolerant tomato *S. pennellii*, a significant increase of the leaf spongy cells size was induced by salinity, with a giant vacuole occupying almost completely the leaf salt-treated cells, which resulted in a higher capacity of Na^+^ accumulation of leaves (Albaladejo et al., 2017).

Other important mechanism for recovering the osmotic homeostasis under salt stress is osmotic adjustment. The plant may use inorganic and organic solutes for reducing osmotic potential (ψ_s_), but the final plant growth will depend on the nature of solutes used. With respect to inorganic solutes, the most important strategy applied by plants for osmotic adjustment under salinity is the accumulation of the saline ions Na^+^ and Cl^-^, which are the cheapest solutes from an energetic point of view (Muñoz-Mayor et al., 2012; Munns et al., 2020). Plants may also accumulate organic solutes to increase their osmotic tolerance against salt stress and maintain their plant growth, which may result in a reversed trade-off between plant growth and tolerance. In previous studies performed in tomatoes, we observed that genotypes exhibiting a high ability to reduce Na^+^ uptake and accumulation within the plant show lower production on the basis of fruit yield (Muñoz-Mayor et al., 2008). The reason is that a lower use of cheap solutes, as are the saline ions coming from the saline substrate or water, increased the biosynthesis of energetically costly organic solutes to maintain the osmotic balance, which implied a growth penalty that negatively reverted on fruit yield. However, it is necessary to take into account that mechanisms to tolerate potentially toxic levels of Na^+^ in the leaf tissues may be valid up to a certain salinity level, but not when the limit of tolerance to cytoplasmic Na^+^ is exceeded. In tolerant genotypes, the Na^+^ uptake was not proportional to external salinity, but was curtailed at high salinities or longer time of exposition to salinity (Estañ et al., 2005).

Although the roots play critical roles in the whole plant physiology, which provides resources to support plant growth, these plant organs have been often overlooked, being breeding mostly focused for quantitative traits as yield. Nevertheless, recent reviews highlight the important role of the belowground plant organ for sustaining plant growth in a degraded environment (Fonseca de Lima et al., 2021; Kohli et al., 2022; Lynch, 2022). Under salt stress, recently Zou et al. (2022) summarized the strategies fulfilled by root growth, among them root system architecture components and directional growth. In addition to improved water and nutrient uptake, deeper rooting system is key to avoid the massive transport of saline ions to the shoot (Cuartero et al., 2010). Thus, if salt tolerance is determined in great part by the plant ability to regulate Na^+^ homeostasis over time, plants with deeper roots would retain more Na^+^ in roots and would reduce Na^+^ transport up to the leaves, displaying better resilience to salt stress.

Advances in the identification of determinants of root system architecture have been mainly achieved on studies on Arabidopsis but the transferability of these results from model plants to crops is still unclear, hinting at the need of investing more research efforts in order to expand this knowledge beyond Arabidopsis. In some crops grown under different environmental conditions, studies with mutants have revealed key roles of roots in maintaining plant fitness in a wide ecological context. Interestingly, Zou et al. (2021) showed that the salt-induced inhibition of rice seminal root growth is mediated by ethylene-jasmonate interaction. Recently, new genetic components related to root development contributing to salinity tolerance in different agricultural species have been identified, such as rice (Patishtan et al., 2018), maize (Zhang et al., 2019), tomato (Wang et al., 2020) and barley (Hazzouri et al., 2018), which will be very useful in the objective of identifying the molecular mechanisms of tolerance and of development of more tolerant crops.

The greatest challenge for the short-term future in agriculture will be to increase crop production under salinity conditions. One approach involves increasing crop yield per unit area through improved agronomic practices and genetic improvement, especially taking into account that domestication has resulted in reduced salt tolerance in many crop species. Thus, different strategies are required, from mapping for salt tolerant genes (Foolad and Jones, 1993) to domestication of salt tolerant varieties genotypes (Wang et al., 2020). Interestingly, these last authors performed a genome-wide association study (GWAS) for root Na^+^/K^+^ ratio in a population consisting of 369 tomato accessions with large natural variations, and they highlighted the important role of SlHAK20 in salt tolerance of tomato. Another approach is the adaptation of alternative stress tolerant crops to marginal environments (Flowers and Colmer, 2008; 2015; Waha et al., 2020; Theeuwen et al., 2022). Furthermore, global agriculture faces the challenge of providing healthy foods despite increasing adverse environmental conditions for growing crops, which requires either to improve the nutritional quality and biodiversity of major crop species or to search new sources of genetic variation adapted to abiotic stressors as underexploited crops (Tian et al., 2022). Talabi et al. (2022) recently defined these crops by using different terms like orphan crops, semidomesticated, underused or forgotten crops, which are generally derived from the state of neglect and abandonment by the scientific community despite their nutritional potential contribution to food. However, these are locally important crop species grown in restricted regions of the world by farmers, in the manner that a plant species may be considered orphan or underexploited crop in one particular region but may not be necessarily considered in the same terms for other regions. One of the most interesting cases is for quinoa, moving out of South America and expanding throughout the world (Alandia et al., 2020).

Most of the reviews published on the current situation and advances achieved during the last years on underexploited crops have been focused on major crops. Thus, Chapman et al. (2022) focus on rice and maize as two of the most widely grown staple crops but also on some crops which could have been described as underutilized but have become mainstream, summarizing in a table the underutilized crops for which reference genomes are available. Talabi et al. (2022) reviewed 13 orphan crops assigned to three categories, cereals and pseudo-cereals, pulses, and oil crops, whose information has been summarized in tables. With regard to the goal of enhancing crop diversity for food security in the scenario of climate uncertainty, Zsögön et al. (2022) showed the first examples of *de novo* domestication, where target genes are modified in order to confer predictable traits of agronomic value, provided in the wild relative of tomato *S. pimpinellifolium* (Li et al., 2018; Zsögön et al., 2018).

This review will summarize advances fulfilled in traditional varieties of tomato based on the following criteria: Adaptation to environmental adverse conditions as well as having high nutritional value, as highlighted in the recent review of Vats et al. (2022) entitled ‘Unexplored nutritive potential of tomato to combat global malnutrition’. Furthermore, its interest is evident as tomato supplies the highest input of nutrients to the world population given its so high *per capita* intake. Here the current situation of two species highly revalorized in last years, quinoa and amaranth, is also summarized. Although information on both species is rapidly increasing in last years, however there is still a great knowledge gap on their tolerance mechanisms and, especially, on the genes involved in abiotic stress tolerance. Finally, it is interesting to highlight that cultivation of both tomato and quinoa and amaranth is extended in the Mediterranean area where, given the scarcity of good quality waters, saline waters are used for irrigation. Furthermore, this area has a well-known record of direct impact of different stressors, which makes more complex the situation of agriculture production.

## Food quality may be enhanced under salt stress

The food quality depends not only on genetic characteristics of crops but also of different factors related with the plant culture systems and environmental conditions (Kyriacou and Rouphael, 2018). Interestingly, one strategy to increase quality may be the plant culture under abiotic stress (Fanciullino et al., 2014). Why may be increased the fruit quality under salt stress? On the one hand, it is known that plants respond to salt stress by increasing the primary metabolites, sugars, amino acids, and organic acids, to recover the osmotic homeostasis (Quinet et al., 2019). Among them, sugars are the most important solutes contributing to the osmotic adjustment, but their use will limit the supply for other physiological functions, such as growth and production. This explains the importance of solute type contributing to osmotic adjustment in the long-term salt tolerance (Munns et al., 2020). Moreover, salinity induces secondary oxidative stress to which plants have to react and secondary metabolites are known antioxidants (Klunklin and Savage, 2017), The two first physiological processes involved in the accumulation of secondary metabolites in fruit are carbon and redox states, both of them are highly susceptible to be altered in stressful conditions (Fanciullino et al., 2014). The plant needs to distribute the photoassimilates from sources (leaves) to sinks (fruits), which will determine the fruit growth under stress and will influence its fruit quality (Osorio et al., 2014). The other process with a major role in accumulation of secondary metabolites is redox state. In response to oxidative stress, plants trigger a plethora of antioxidant mechanisms, including those of non-enzymatic nature as accumulation of carotenoids and tocopherols (Mittler et al., 2022). In sum, an excellent way to increase secondary metabolites in fruit, which are very important as nutraceuticals for the human diet, might be growing crops under abiotic stress conditions.

But the problem is very much complex due to the different factors involved in plant response to stress and, therefore, to the many possibilities resulting of these situations. Thus, final plant growth and fruit quality would be determined by culture conditions, and cultivation systems, as field and greenhouse, as well as environmental conditions in different years, areas and seasons (Asensio et al., 2019; Moles et al., 2019). But one of the most important factors are the geographic areas, and major differences are being found in those subjected to multiple environmental stressors such as the Mediterranean area (Balestrini et al., 2021). The problem is that the multiple effects may be very different to those initially expected from the individual effects or even to those combining only some of them (Rivero et al., 2022; Zandalinas and Mittler, 2022). However, some combinations seem to display common effects, which may be very important for future strategies to improve agriculture performance in multiple abiotic stresses. This was the case for a combination of six different abiotic stresses (heat, high light, salinity, acidity, cadmium and oxidative stress induced by the herbicide paraquat), where Zandalinas et al. (2021) observed that genes involved in reactive oxygen species (ROS) homeostasis could be key factors for upholding crop production under stressing natural conditions.

**Figure 1:**
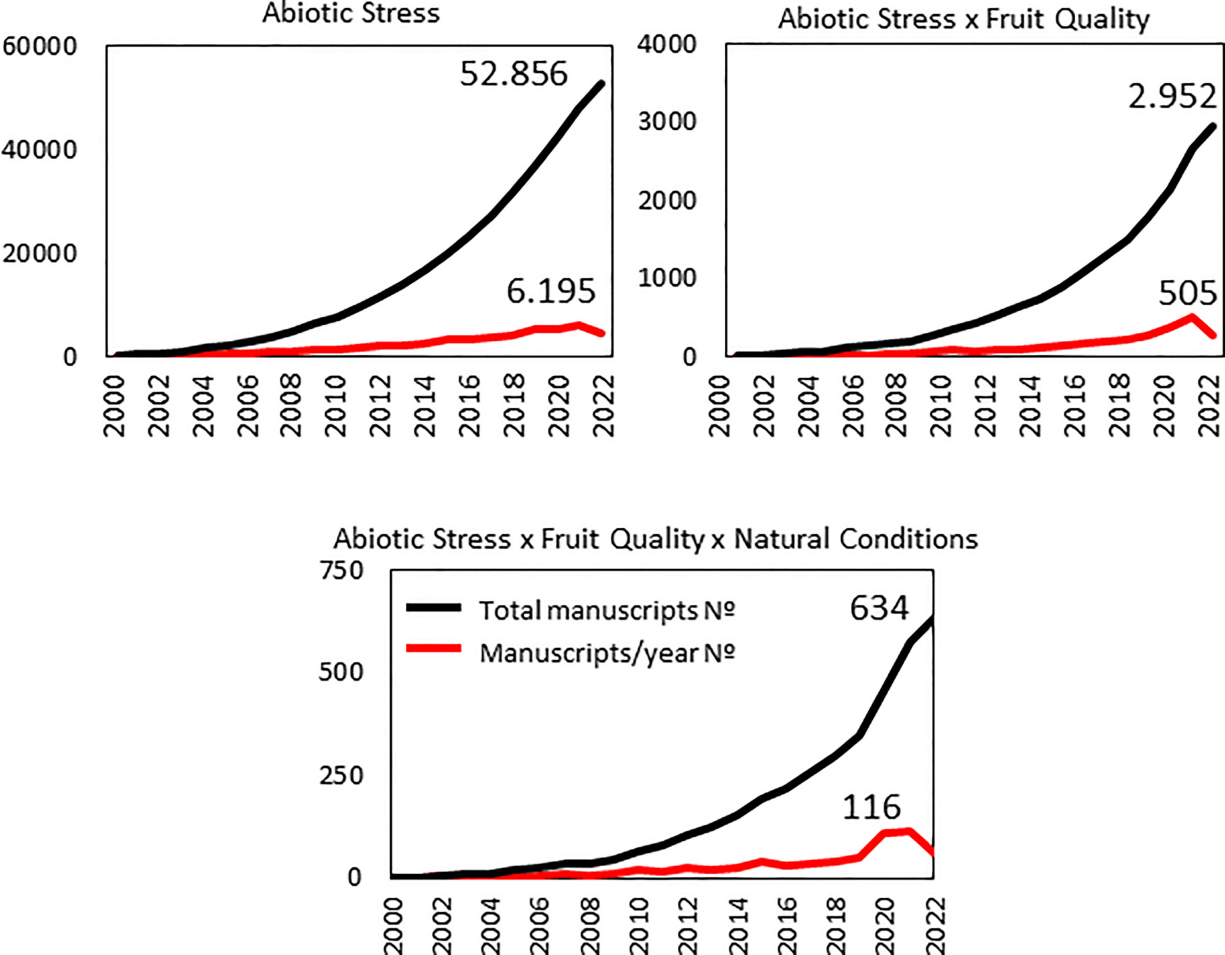
Number of manuscripts published between 2000 and 2022 years within the topics ‘Abiotic stress’, ‘Abiotic stress x Fruit quality’, and ‘Abiotic stress x Fruit quality x Natural conditions’. Source: Web of Science. It is evident the important reduction of published articles for ‘Abiotic stress x Fruit quality’, and still more when searching is restricted to ‘Abiotic stress x Fruit quality x Natural conditions’.

In such situations, it would be necessary to perform frequent and multiple assays in natural conditions, as they are relatively scarce when compared with the total number of research works published on abiotic stress and fruit quality. Thus, we performed a literature search in the Web of Science for the general topic ‘Abiotic Stress’ and found more than 50000 manuscripts in 2000-2022 (**Figure 1**). This huge figure is dramatically reduced for the interaction ‘Abiotic stress x Fruit quality’ (2952 papers, less than 6% of the previous amount). The importance of this interaction seems to have grasped the attention of the research community very recently, as the percentage of manuscripts published during last year on the topic increased up to 8.2%. Finally, the knowledge reached and published about the ‘Abiotic stress x Fruit quality’ interaction but under ‘natural conditions’, which so often occurs in nature, are insufficient according to the so low number of publications we have found, just 634 manuscripts in the last 22 years. However, it is also interesting to remark the increased percentage of manuscripts in last year (23%) compared with just ‘Abiotic stress x Fruit quality’, which suggests that generation of fruit-bearing varieties with high tolerance to multiple stressors might be achieved in a relatively short time period, being very relevant in the projected increasing adverse environmental conditions of climate change (**Figure 1**).

In sum, the crop responses to abiotic stresses and the accumulation of health-promoting compounds under stress are crucial in a scenario of climate change. Unfortunately, there is still an important gap of knowledge for achieving the goal of producing more food with fewer resources, in order to achieve the zero hunger Sustainable Development Goal (SDG2) in a near future. From an agronomic standpoint, it is highly and urgently needed more advances focused on increasing quality food without reducing production, which seems to be possible by imposing controlled abiotic stress through the plant life cycle.

## Use of tomato traditional varieties as genetic resources

Tomato (*Solanum lycopersicum* L.) is one of the most consumed and produced fruit-bearing species worldwide assessed by both the cultivated surface and the importance of the fruit in nutritional terms, particularly taking into account that tomato supplies the highest input of nutrients to the world population given its so high *per capita* intake. But what changes in the fruit composition might be occurring as consequence of salt stress? Firstly, some minerals seem to be affecting fruit nutritional quality under stress. The reasons are evident since the fruit growth depends on the ability to reduce fruit osmotic potential (ψ_s_), where inorganic solutes have an important role under salt stress. Thus, Bolarin et al. (2001) demonstrated that the tolerant accession of the wild-related tomato species *S. pimpinellifolium* did not reduce fruit weight under salt stress, contrarily to cultivated tomato, due to the ability of their fruits to accumulate inorganic solutes during the rapid growth phase. Under salinity, in addition to the saline ions coming from substrate or water, another important inorganic solute is K^+^, which is one of the essential elements required for plant growth. Thus, alterations in K^+^ can disturb the osmotic balance, the function of stomata and that of some key metabolic enzymes (Nieves-Cordones et al., 2019). Moreover, K^+^ has been proposed to be associated with sucrose unloading from the phloem into the fruit. Increase of K^+^ use efficiency in crops is especially required for future low-input agriculture within a context of rising impact of abiotic stress and an increasing population (Ródenas et al., 2021).

In the particular case of salinity, a nutritional imbalance generally occurs by reduction of the levels of essential mineral nutrients such as Ca^2+^, Mg^2+^, P and Fe (Loudari et al, 2020; Agius et al., 2022). Calcium is an essential element for plant growth as well as for fruit development (Kudla et al., 2018), and Ca^2+^ deficiency symptoms are very frequent in tomato cultured under salt stress. We demonstrated the important role of the tomato Ca^2+^ signaling gene *Calcineurin B–Like Protein 10 (SlCBL10*) in fruit production and quality, since a very high incidence of the physiological disorder known as blossom end rot (BER) was detected in fruits of the *SlCBL10* null mutant (Egea et al., 2018a). The symptoms observed were fruit growth cessation and the fruit area affected by Ca^2+^ deficiency, which starts growing from the apical zone of the fruit, inducing cell necrosis and becoming brownish. Although the studies performed to elucidate the basis of this physiopathy are numerous, however the problem is unsolved (Topcu et al., 2022), and more information on the specific roles of altered Ca^2+^ homeostasis among different cellular compartments is lacking.

The primary metabolites are essential in the quality of tomato fruits. One trait associated to sugars is soluble solids content (SSC). For a long time, SSC has been used as a standard fruit quality trait, serving as the overall determinant of tomato fruit organoleptic quality. SSC measured by refractometry as Brix proportion of all dissolved solids in water does not allow to know exactly the predominant solutes in the fruit, but this trait is especially important in tomatoes for the processing industry (Beckles, 2012). There are numerous studies about the changes induced by abiotic stress in sugars, organic acids and amino acids (Quinet et al., 2019 and references therein). Within this last group of metabolites, one of the processes involved in abiotic stress tolerance is the GABA shunt, allowing the use of amino acids as alternative substrates for mitochondrial respiration when the sugar supply is insufficient due to photosynthesis reduced by stress (Batista-Silva et al., 2019). Interestingly, genes linked to GABA shunt were induced by drought tolerance in the wild tomato species *S. pennellii* (Egea et al., 2018b). The activation of the GABA-shunt stimulates the conversion of Glu into succinate ready to be used by the TCA cycle, so contributing to maintain the C/N balance in the wild species under drought conditions. Secondary metabolites, carotenoids and tocopherols are among the main components found in tomato fruit with particularly important nutritional properties (Raiola et al., 2015; Sun et al., 2022). Under stress, both metabolites play crucial roles in photosynthesis and photoprotection; they are essential antioxidants exclusively produced in plastids during tomato fruit ripening (Almeida et al., 2011; Enfissi et al., 2017; Llorente et al., 2017; Sun et al., 2022). One of the most interesting questions to solve in this aspect is understanding the metabolic network of isoprenoids biosynthesis, given the connection among chlorophylls, carotenoids and tocopherols (Almeida et al., 2015).

However, the resulting picture of fruit quality is rather complex due to diverse factors influencing the final quality of fruits. Thus, Moles et al. (2019) studied the salinity response to different concentrations of NaCl of three tomato landraces (Ciettaicale, Linosa and Corleone) from Southern Italy, and one commercial cultivar (UC-82B), and they observe that the combination of moderate/high salt concentrations with low light irradiance differently affected yield and metabolism of the tomato genotypes. On the other hand, Asensio et al. (2019) observed different effects of genotype, location and agronomic conditions on the nutritional quality and evaluation of consumer preferences in several Spanish traditional tomato varieties. Their results confirmed that the fruit quality of tomato “Rosa of Barbastro” could be significantly modified with the agronomic conditions. During years of research evidence arose showing that the higher the abiotic stress degree, the higher food quality and, therefore, it might be more interesting to perform multiple assays in arid- and semi-arid zones, as Mediterranean areas, exposed to multiple stress. One of the main stressors of arid and semi-arid areas is light, where photoreceptors phytochromes (PHYs) play a central role in controlling fruit physiology, and together with PHYTOCHROME-INTERACTING FACTORs (PIFs), they greatly affect tomato fruit yield and fruit quality (Bianchetti et al., 2018; Gramegna et al., 2019; Rosado et al., 2019; Alves et al., 2020). In this context, the tomato traditional varieties will allow performing deeper studies on the metabolic and molecular responses of tomato to abiotic stress and evolution of tomato fruit quality, especially in world areas or regions where deleterious effects of climate change are expected to occur sooner and in a more intense degree. But what are the advances achieved until now by growing tomatoes in Mediterranean areas exposed to multiple stress? In Italy, the recovery of traditional varieties is important, as it has occurred with the San Marzano varieties (Loiudice et al., 1995), which grow best in warm climates with long-term Mediterranean-type summers (Tranchida-Lombardo et al., 2018), and play a key role in conservation of such genetic diversity. In Spain, some very interesting traditional varieties were selected on the Mediterranean area because of their ability to maintain long shelf-life under drought conditions (Conesa et al., 2020). These promising results suggest that a very attractive strategy is to incorporate underutilized crops for enhancing simultaneously tolerance and quality, allowing us a higher understanding of the phenotypic diversity in an environmental context.

### Promising variation sources to obtain healthy foods in the Spanish Southeast

**Figure 2:**
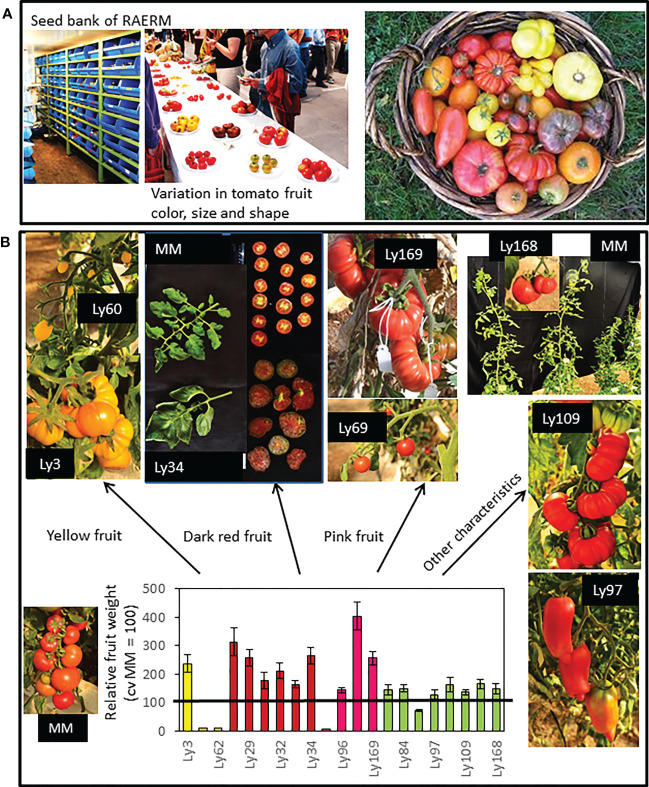
**(A)** Seed bank and a representative sample of the variation in fruit size, color and shape of tomato traditional varieties collected by the Agro-Ecology Network of the Region of Murcia (RAERM). **(B)** Representative pictures of varieties belonging to groups of tomatoes classified by the fruit size, fruit color and other characteristics. In the graphic the relative fruit weights with regard to commercial cv Moneymaker (MM, used as reference) are included. Sources: Flores et al., 2022. Recuperación de variedades tradicionales de tomate de alta calidad de fruto para su cultivo en condiciones de la cuenca mediterránea. Avances en maduración y poscosecha de frutas y hortalizas (E. Arias, S. Remón, R. Oria Eds., Servicio de Publicaciones-Universidad de Zaragoza, ISBN: 978-84-18321-41-2), pags 51-60. Meza et al., 2020. Traditional tomato varieties improve fruit quality without affecting fruit yield under moderate salt stress. Front. Plant Sci. 11:587754. doi: 10.3389/fpls.2020.587754. Massaretto et al., 2018. Recovering tomato landraces to simultaneously improve fruit yield and nutritional quality against salt stress. Front. Plant Sci. 9:1778. doi: 10.3389/fpls.2018.01778.

Currently, we are beginning to explore the possibilities of one new collection of tomato traditional varieties from the Spanish Southeast area, where the predominant climate is arid-semiarid, with mild winters and dry and very hot summers, and very scarce rainfall levels (< 300 mm per year). But at the same time this area is one of the main ones for tomato production in the world. The adverse conditions proper of the area, featured by high temperatures that induce high evaporation rates leading to water scarcity, are predicted to worsen according to climate change projections (IPCC, 2022). Considering also that groundwater resources are being depleted at an alarming pace farmers in this area are obliged to make use of saline waters for irrigation. This situation is extremely adverse for agricultural production. This Spanish Southeast area is the cradle of a suite of tomato landraces particularly adapted to these harsh environmental conditions, which has been gathered and registered by the Agro-Ecology Network of the Region of Murcia (RAERM) (Egea-Fernandez et al., 2015). In our lab, one of our goals is to understand the biochemical and metabolic diversity of the traditional varieties’ collection in order to assess their aptitude for their inclusion in breeding programs to obtain tolerant genotypes to abiotic stress and simultaneously with enhanced quality of their fruits. Our previous results reveal their great value as plant materials targeted at developing future varieties able to thrive in severe environmental conditions. Moreover, it is very interesting to highlight the important variation in fruit morphology, including fruit color, shape and size of fruit, as well as other vegetative traits such as vegetative biomass, leaf size, plant height, etc. (**Figure 2**). Representative photographs of varieties belonging to groups classified by fruit color are shown, including yellow, dark red and pink varieties, an important trait very much related to the accumulation of different secondary metabolites. From the point of view of salt stress tolerance, it is very interesting to come up with varieties with large fruit size since these are generally more affected by this stress (Albert et al., 2016). The whole collection is being compared to commercial cv. Moneymaker (MM), which is an indetermined cultivar, with mid-size and round-shaped fruits containing two locules, of high production in optimal conditions rendering homogenous fruits in shape and color (**Figure 2**).

At the agronomic level, preliminary results confirm the interest of these materials for growing tomatoes under abiotic stress. Thus, the fruit yields of two varieties with very different characteristics, named Tomate Pimiento” (TP) and “Muchamiel Aperado” (MA) were not affected at 50 mM NaCl (Meza et al., 2020). TP fruits are mid-sized although slightly bigger than those of cv MM used as reference, but their so different shape (elongated and similar to bell peppers compared to round-shaped type in MM), while MA present fruits of larger size (more than two-fold bigger in weight terms compared with MM) of ‘Muchamiel’ typology but with pear/oxheart shape with weak ribs. Taking into account that tomato varieties of large fruit size are generally more affected by stress (Albert et al., 2016), the sustainable fruit yield found in MA subjected to moderate salt stress makes this variety very attractive from a point of view of salinity tolerance. What is more, MA exhibited a similar response under water deficit conditions and high temperatures, where reduction of average fruit weight because of these stresses is notably lower than MM (Ballesta et al., 2022).

The effect induced by salinity on the production may vary with the position of trusses in the tomato varieties of indeterminate growth, increasing their intensity in superior trusses, developed later and subjected to longer periods of salt treatment when fruits are harvested. Interestingly, similar fruit yields for the first, second and third trusses of the assessed tomato traditional varieties were found at moderate salinity (Meza et al., 2020). Another factor to take into account is the salt concentration of the irrigation water, since the salt tolerance mechanisms required to face the stress may be very different depending on the stress level (Albaladejo et al., 2017). In a study performed at high salt stress level (100 mM NaCl), we studied contrasting traditional varieties regarding fruit size to know the influence of this trait at this salinity level: Negro Yeste (NY) with fruits of small size Pera type (cherry), and Verdal (V) with big size fruits exhibiting a high locule number (Muchamiel type) (Massaretto et al., 2018). Interestingly, not only the small NY fruits showed higher salt tolerance than MM regarding yield but also the big fruits of V, which was mainly reflected in the fruit number. Although more studies are necessary, we wish to highlight that these landraces may be very useful to uphold productivity under abiotic stress. Next, we summarize the different quality improvements induced by cultivation in stressful conditions in the tomato traditional varieties we have studied.

### Fruits with enhanced mineral nutrients content

**Figure 3:**
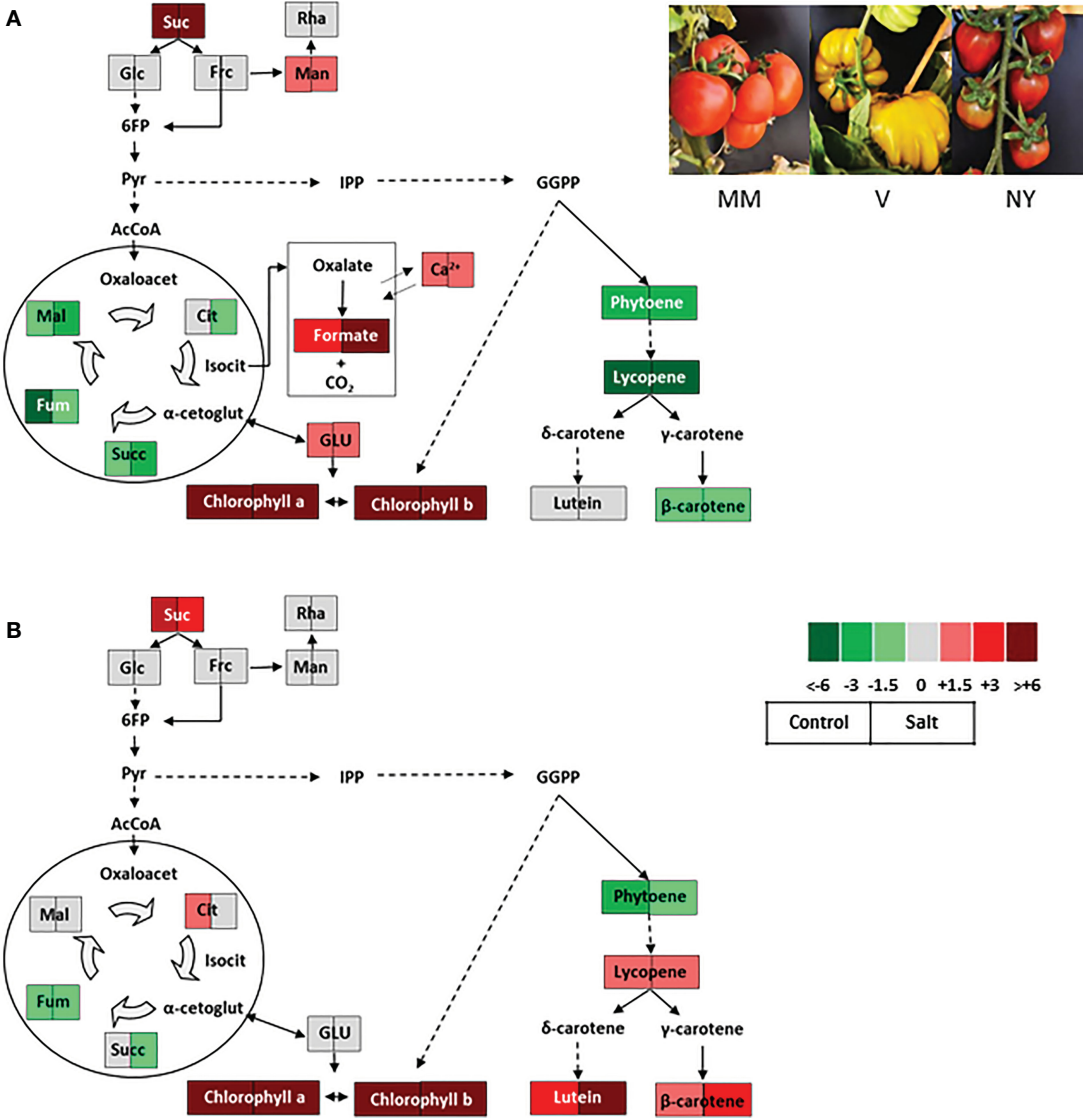
Contrasting metabolic profiles in ripe fruits of two traditional tomato varieties coming from the Spanish Southeast area and belonging to the RAERM collection. Schematic representations of the metabolic changes occurring in ripe fruits of varieties **(A)** Verdal **(V)** and **(B)** Negro Yeste (NY) from plants grown without stress (control) and with salt stress (100 mM NaCl during 70 days). Data were normalized to cv Moneymaker (MM). Color scale is used to display the different amount of metabolite in terms of fold-change relative to the level in the appropriate control. Suc, sucrose; Glc, glucose; Frc, fructose; Man, mannose; L-asc, L-ascorbic acid; 6FP, fructose-6-phosphate; Pyr, pyruvate; IPP, isopentenyl diphosphate; GGPP, geranylgeranyl diphosphate; AcCoA, acetylCoA; Oxaloacet, oxaloacetate; Cit, citrate; Isocit, isocitrate; α-cetoglut, α-cetoglutarate; Succ, succinate; Fum, fumarate; Mal, malate; GLU, glutamate. Adapted from Figures 7, 8 of Massaretto el al. 2018. Recovering tomato landraces to simultaneously improve fruit yield and nutritional quality against salt stress. Front. Plant Sci. 9:1778. doi: 10.3389/fpls.2018.01778.

A primary trait in tomato fruit quality is mineral composition, being the studies focused in it rather scarce to date. When studying the salt stress responses of V variety, we observed that this variety presented high Ca^2+^ levels in ripe fruits compared with MM (**Figure 3**) (Massaretto et al., 2018), which is very interesting given the evident importance of Ca^2+^ in salt tolerance but also in fruit quality (Egea et al., 2018a). It is also interesting to point out that the ripe fruits of this variety also present high levels of formate (**Figure 3**), which may partipate in biosynthesis of numerous compounds, in energy metabolism and in signal transduction pathways related to stress response (Lou et al., 2016). This traditional variety (V) may be a very interesting resource to advance in the knowledge of the formate metabolism, a health-promoting component scarcely studied to date in fruit metabolism.

We have already mentioned the so negative impact of BER in fruit quality and therefore fruit production, which is associated with altered Ca^2+^ homeostasis. Recently, Topcu et al. (2022) summarized the problems caused by this physiopathy in tomato, remarking that the identification of genes involved in BER development would result in a deeper understanding of the molecular bases of this disorder. To approach this goal it is necessary to select adequate materials, and some of the tomato traditional varieties of our collection could be very good candidates. Thus, in our multi-stressor Mediterranean growth conditions, we have observed an outstanding result very much related to productivity of marketable fruits: the so high amount of MM fruits affected by BER compared with some landraces; while in the former 40% of fruits were affected with respect to the total number harvested, in Murciano Rojo (MR), the already mentioned MA, and Tomate de Almaciles (TA) traditional varieties the BER impact hardly attains 5% under heat and water stresses (Ballesta et al., 2022). Another interesting genotype for the study of the processes involved in BER development is the mentioned V, given its so high Ca^2+^ levels in ripe fruits (Massaretto et al., 2018).

### Varieties selected for primary metabolites

As we have already mentioned, a key fruit quality trait is SSC, which serves as an overall determinant of tomato fruit organoleptic quality. In the tomato traditional varieties analyzed till now one improved trait induced by salt stress is precisely SSC, as observed in the NY and V varieties, compared with MM, when the plants were grown at high salt level (100 mM NaCl) (Massaretto et al., 2018). Interestingly, even increasing fruit SSC level was observed in TP and MA traditional varieties grown at moderate salt level (50 mM NaCl) (Meza et al., 2020), which revalorizes the use of these for processing tomato products. Furthermore, one interesting characteristic of these SSC increases in the tested traditional varieties is that they are the result of a *per se* accumulation of sugars in the fruits of plants grown in salt stress and not a consequence of a concentration effect caused by water reduced content in the fruit (Albert et al., 2016).

Primary metabolites are critical for recovery of the metabolic homeostasis under stress but they are even more crucial in improvement of fruit quality. Regarding sugars, the first major metabolic change found in traditional varieties was sucrose accumulation in red ripe fruits, both at moderate salt level, when no production loss was observed with salinity (Meza et al., 2020), and when fruit yield was reduced at higher salt level (Massaretto et al., 2018). These results imply that fruit quality is linked to a greater ability to accumulate sucrose in landraces. However, no significant increases were generally found in organic acids, even the levels were reduced with regard to MM in some varieties (**Figure 3**). Regarding amino acids, the most interesting variety selected until now is TP because of their high constitutive levels found in its fruits, including the three major free amino acids present in tomato fruit, GABA, Glu and Gln, which even increased under salinity (Meza et al., 2020). Increased amino acids levels in traditional varieties compared with MM were observed not only under salt stress but also under other abiotic stresses. Thus, it is remarkable the rising levels of amino acids in fruits of MR, MA and TA local accessions compared with MM when plants were grown with water deficit in summer season, where very high temperatures are attained (> 40°C at midday) (Ballesta et al., 2022).

### Selection for secondary metabolites: Carotenoids and tocopherols

The improved fruit quality of some tomato traditional varieties was also associated to high carotenoids content, as the NY fruits contain high levels of β-carotene and lutein in salt-treated fruits (Massaretto et al., 2018). For the same genotype, different changes may be associated in function of the type of abiotic stress. Thus, while water deficit seems to lead to an increase in carotenoids (Albert et al., 2016), high temperatures generally reduce carotenoid contents, especially if the treatment is imposed during advanced stages of fruit development (Almeida et al., 2021). We studied the agronomic response and fruit quality effects of selected traditional varieties grown in high temperature (HT) conditions during the reproductive period. In addition to HT, we also studied the response of these tomato landraces to a reduction of the water regime, up to a rather high level (60% reduction compared with standard cultivation), and, therefore, the effects on fruit quality and yield will be the result of combining both stresses, HT plus water deficit (WD) (Ballesta et al., 2022). Interestingly, no significant differences in carotenoids levels were found in HT+WD vs HT growth conditions in fruits from one of the varieties, TA, and these were very low in another, MA, showing the ability of some tomato genotypes to uphold fruit carotenoids in plants exposed to HT+WD. On the contrary, another tomato landrace, MR, exhibited a very high content in carotenoids in ripen fruits of plants subjected to HT compared with the rest, including MM, but this dramatically descended in HT+WD growth conditions.

The other major group of secondary metabolites from the tomato fruit nutritional quality perspective are tocopherols, and we observed that their contents in tomato fruits increased under salt and water stress in some traditional varieties such as TP, MA, MR and TA (Meza et al., 2020; Ballesta et al., 2022). Interestingly, the α-tocopherol content rose more than 50% in red ripe fruits of TP and MA under moderate salinity, although this increase was not accompanied by enhanced carotenoids. With regard to fruits of plants from traditional varieties grown under HT+WD conditions, MR, MA and TA, the content in such metabolite is notably superior compared with MM (Ballesta et al., 2022).

Our previous results show that the main secondary metabolites increasing in the selected tomato varieties grown at high salt level (100 mM NaCl) were carotenoids (Massaretto et al., 2018), whereas when a lower intensity of salt stress treatment (50 mM NaCl) was applied then it was α-tocopherol the secondary metabolite that significantly increased (Meza et al., 2020). Is this behavior due to the genetic background or to the different salt stress levels applied? Which is playing the predominant role? This dilemma has to be solved in future studies, that will be focused in investigating the fluxes of isoprenoids pathways under salt stress. As it has been previously pointed out, heat stress may negatively affect tomato fruit quality but interestingly, MA landrace exhibited a remarkable significant high α-tocopherol content compared with MM in fruits of plants subjected to heat stress (HT), and this increase is even higher when the plants were submitted also to water deficit (HT + WD) (Ballesta et al., 2022). In any case, intake of tomato fruits with increasing levels of carotenoids and α-tocopherol have undeniably beneficial effects for human health (Choi et al., 2014; Saini et al., 2017). Therefore, tomato landraces constitute an excellent genetic resource to reach such benefits for human diet in a context of agricultural production faced with harsh environmental conditions due to climate change.

Finally, it would be interesting to elucidate the mechanisms by which fruits redirect the metabolic flux mainly towards one of the pathways and, therefore, predominant accumulation of one or other metabolite occurs. This metabolic crosstalk occurs for carotenoids, tocopherols, and chlorophylls, which share a common precursor, geranylgeranyl diphosphate (GGPP), coming from the MEP pathway (Egea et al., 2022). The fruits of MA and TP from plants under moderate salt stress showed different responses, as an inverse relationship between α-tocopherol and carotenoids was observed in the first, whereas TP showed an increase in α-tocopherol while it presented similar carotenoid levels (Meza et al., 2020). The high α-tocopherol found in TP may be due to the high content detected of amino acid tyrosine, key component of the shikimate pathway that renders the other precursor of tocopherols, while in MA a trade-off between this metabolite and carotenoids is occurring. A very interesting example that highlights the interest to pursue these studies with tomato landraces is the case of V, where the carotenoid biosynthesis pathway normally functions in leaves and immature fruits but it is disrupted in ripe fruits (**Figure 3**) (Massaretto et al., 2018). In conclusion, our collection of tomato traditional varieties constitutes a very attractive source of genetic variation in tomato. Taking into account the multiple stressors found in this region (Spanish Southeast), with very hot summers and very scarce hydric resources that forces farmers to use saline waters for irrigation, they constitute a rich genetic reservoir for breeding focused in improving tomato plant tolerance to abiotic stresses and tomato fruit quality in a scenario of climate change.

## Use of Amaranthaceae family: Quinoa and Amaranth

Quinoa (*Chenopodium quinoa* Willd.) and Amaranth (*Amaranthus* spp.) come from the Andean region of South America. Both belong to the Amaranthaceae family, which comprises the highest proportion of salt-tolerant halophytic species. Within amaranth, there are grain and leaf species, being *Amaranthus cruentus, A. caudatus*, and *A. hypochondriacus* the most important grain sources, and the black-seeded amaranth species *A. tricolor*, *A. blitum* and *A. dubius* are cultivated to harvest their leaves (Meza et al., 2022). The three grain species mentioned above are also being used as leafy vegetables in some countries from sub-Saharan Africa and Asia (Ma et al., 2021), so distinction between amaranth species for grain and for leaves is based in cultural rather than scientific issues.

The seeds of quinoa and amaranth have received much attention in recent years because of their very high nutritional value (Tang and Tsao, 2017; Angeli et al., 2020; Meza et al., 2022). From the perspective of their cultivation in abiotic stress conditions, the high genetic variability of quinoa and amaranth constitutes a great advantage, as they come from tropical to temperate climatic regions, included semi-arid and arid areas affected by multiple stressors. Thus, cultivation of these species has been gaining attention in other regions of the world very different from their original ones (Alandia et al., 2020; Rodríguez et al., 2020). One of the problems limiting advances in these underutilized species is that their genomics studies are rather scarce to date, so genetic breeding is, in part, constrained by limited genomic resources (Chapman et al., 2022). However, significant progress has been made in quinoa, with a significant boost in research, where the (tetraploid) quinoa genome has been made available, and recognition of its agro-economic importance, with outstanding increases, bigger than 500%, in the world surface devoted to its cultivation from 1960s (Jarvis et al., 2017). In amaranth, genomes of *A. hypochondriacus* and *A. cruentus* are already available (Lightfoot et al., 2017; Ma et al. (2021). The information enclosed by these genomes have much to offer in terms of nutritional value of the species and its cultivation in adverse environmental conditions.

From the point of view of quality, quinoa and amaranth grains have been recognized as complete foods due to their excellent composition of essential nutrients, especially regarding amino acids balance and different phytochemical functional compounds (Tang and Tsao, 2017; Angeli et al., 2020; Meza et al., 2022). Among amino acids of major interest for nutrition, the contents of lysine, threonine, and tryptophan stand out, as they are much higher than those found in common cereals, all of them deficient in these essential nutrients (Berghofer and Schoenlechner, 2002). Seeds of quinoa and amaranth are also excellent sources of antioxidants, with their tocopherols and carotenoids contributing to the antioxidant activity, and also they are free of gluten, so they may be recommened for patients with coeliac disease. In addition, their consumption has another set of health benefits like prevention of cardiovascular and other chronic diseases, cancer, obesity and diabetes (Tang and Tsao, 2017). For a revision of proteins quality of amaranth seeds see Meza et al. (2022, and references therein). Food production derived from quinoa and amaranth grains is forever increasing, being the advances attained very important not only in traditional food products but also in novel industrialized food products (Angeli et al., 2020; Meza et al., 2022). In sum, searching for sources of quinoa and amaranth rich in nutritive and health-promoting components are necessary to advance in this important food research area, in their ever increasing multiple uses for development of healthy human foods.

Quinoa and amaranth are regarded as attractive crops that might assist to sustain food security in the current scenario of climate change (Tovar et al., 2020). In spite of numerous studies published for quinoa in recent years describing the effects of abiotic stress on its growth, much more research is necessary to elucidate how diverse genotypes respond to different environmental situations (Hinojosa et al., 2018; Manaa et al., 2019; Talabi et al., 2022). From the point of view of adaptation processes involved in tolerance to abiotic stress, it is very interesting the different anatomic characteristics of quinoa and amaranth plants. In quinoa plants, the specialized epidermal bladder cells (EBCs) that have a key role as salt sinks for external sequestration of Na^+^ stand out (Böhm et al., 2018). But EBCs may be not only storage sites for excess of saline ions in leaf but also for the storage of water and different metabolites (Bazihizina et al., 2022). The leaf structure of amaranth is the typical Kranz anatomy of C4 plants, with the bundle sheath (BS) cells containing centripetally located chloroplasts and a layer of mesophyll cells surrounding these BS cells (Ueno, 2001). But not only important differences are found between species but even within species. For example, varieties with includer and excluder characters have been identified in quinoa despite the fact that leaves of this species are characterized by presenting EBCs. Thus, Cai and Gao (2020) used five contrasting quinoa cultivars belonging to highland ecotypes and observed lower Na^+^ content in leaf than in root, which suggests that these quinoa cultivars are Na^+^ excluders.

For amaranth, there are almost no studies on the salt tolerance and tolerance mechanisms operating in this species and the scarce results up to date are not sufficiently robust. Thus, we performed a comparative study to investigate the mechanisms used by amaranth and quinoa when a not very high salt level is applied (100 mM NaCl), which warrants seed production. Plant growth was not affected by salinity at mid-term (20 days of salt treatment) in both species (Estrada et al., 2021). Contrarily, Gandonou et al. (2018) studied the salt response of amaranth plants when applying 0, 30 or 90 mM NaCl during 20 days, and they observed that salt stress induced a significant reduction in shoot growth of amaranth plants and, therefore no high degree of salt tolerance was determined in these accessions. These diverging results might be due to the diverse genetic backgrounds of amaranth used in each study, *A. caudatus* and *A. cruentus*, respectively (Gandonou et al., 2018; Estrada et al., 2021). The responses may also differ when these species are grown in conditions where multiple stressors are present, as it occurs in the Mediterranean area, as effects of different stresses combined may be synergistic and antagonistic (Zandalinas and Mittler, 2022).

### Selection of amaranth and quinoa varieties for cultivation in the Spanish Southeast

To achieve sustainably yield rises with improved nutritional quality and simultaneously maintaining biodiversity, proof-of-concept cultivation assays are required in selected crops and this is a challenging goal, which involves trials with different species in diversified agricultural systems subjected to multiple environmental stressors. The Mediterranean area is a highlighted region because of its high-yield agricultural production but it is also one very much affected by the adverse effects of climate change, and climatic projections predict more intense deleterious effects on such production in different crops. The Spanish Southeast has a long tradition of outstanding crop production as well as high investment in agronomic research and plant breeding. In particular, the Region of Murcia, located in such area, is the site of many research institutions and companies from the agro-food sector with a significant impact on agriculture. At the same time, it is one of the Spanish regions enduring one of highest negative impacts of climate change, with very high summer temperatures and very high light radiation intensities, consequently with high evaporation rates that dramatically diminish hydric resources, inducing salinization of soils because farmers too often are obliged to make use of low-quality water for irrigation.

**Figure 4:**
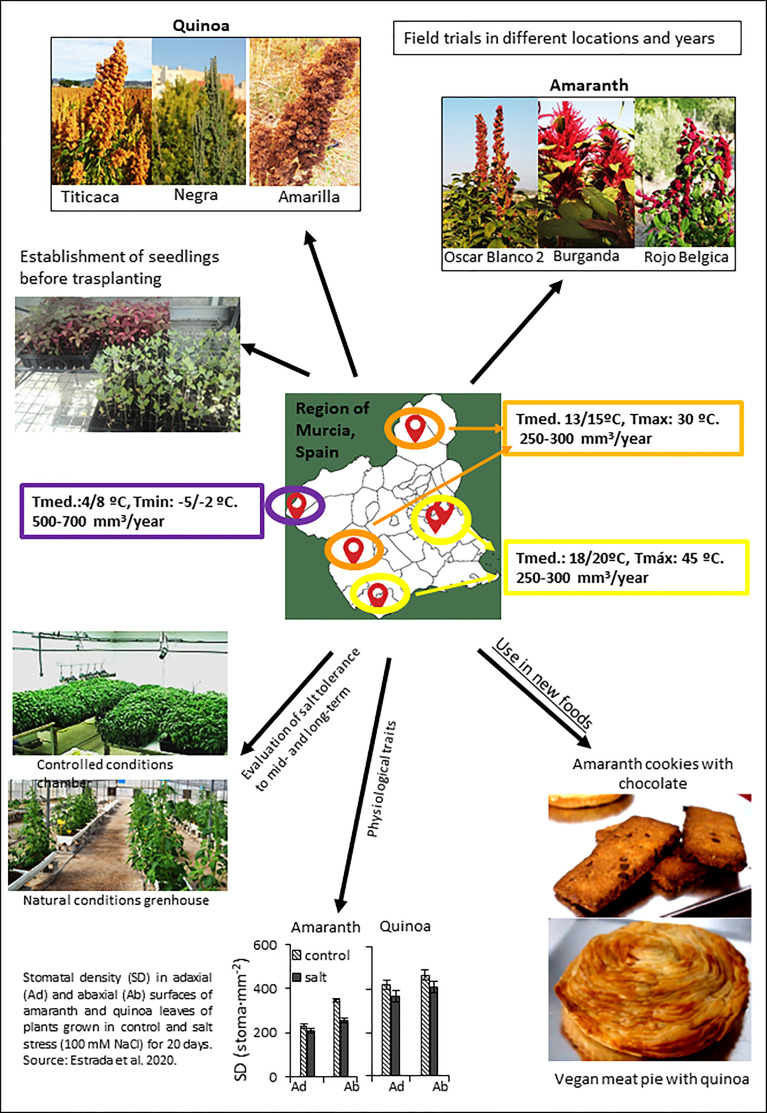
A schematic representation of activities carried out within the project “Agro-Ecological Innovation Observatory against Climate Change” (AEI-CC), focused in selection of quinoa and amaranth genotypes to be grown in the Spanish Southeast (Region of Murcia) with potential to climate change adaptation as well as exhibiting good organoleptic and nutritional qualities. Varieties with very different characteristics are being evaluated in field trials in different locations (see map) to optimize the culture conditions and to achieve maximum seed yield and seed quality. In addition, salt tolerance is also being evaluated to select the best varieties under stress, by simplifying the selection of new varieties by means of analyzing physiological traits. Finally, the elaboration of new products based on amaranth and quinoa in food industries is another objective of the project.

The project “Agro-Ecological Innovation Observatory against Climate Change” (AEI-CC) addresses these challenges in agriculture in Southeastern Spain by approaching the study of farming promising crops such as quinoa and amaranth, exploring their potential to climate change adaptation as well as analyzing their organoleptic and nutritional qualities. This is the first project of this Spanish region that main funding comes from the “Rural development 2014-2020 Plan for Operational Groups”. A schematic representation of activities carried out within the project “Observatory of Agro-Ecological Innovation against Climate Change” (AEI-CC), directed to select quinoa and amaranth genotypes in the Spanish Southeast with potential to climate change adaptation as well as with good organoleptic and nutritional qualities, is shown in the **Figure 4**. What is particularly noteworthy about the project is that it involves a collaborative effort among scientists and non-academic institutions such as Small and Medium-sized Enterprises (SMEs), joining forces for the success of it. Around half of its partners comprise those who are actively growing quinoa and amaranth for selection of the best varieties. A crucial aspect for **AEI-CC**’s success is its connections with other initiatives that focus on characterizing natural diversity of the Amaranthaceae family. Here it is summarized the most important advances achieved to date, including field growth evaluations of the selected amaranth and quinoa varieties conducted in different locations of the Spanish Region of Murcia, the new foods which are being prepared with them, as well as the research on their agronomic, physiological and molecular responses to salt stress.

Under the framework of the AEI-CC project, diverse cultivation assays have been initiated with different varieties of quinoa (*Chenopodium quinoa*) and amaranth (*Amaranthus caudatus* and *A. hypochondriacus*). The three species are characterized by their great heterogeneity, with varieties tolerant to extreme climatic and edaphic conditions, which can be grown with few inputs and exhibit an extraordinary nutritional quality, with functional compounds of great interest for human and animal health (Egea Fernandez et al., 2015). Both quinoa and amaranth have been part of the Andean highlands diet during thousands of years. Its consumption is similar to that of cereals, but the absence of gluten makes them suitable for celiac patients.

The general objective of the assays is to analyze if different varieties of quinoa and amaranth are able for optimal growth in the agro-climatic conditions of the Region of Murcia in Southeast Spain, and if they are good alternatives to the current dominant crops, in a climate change scenario. The assays with quinoa date back to 2015, with the variety Salcedo-INIA. This variety, after sowing it during three years at different seasons, was discarded because of its inability to endure the high summer temperatures proper of the area. From 2018 the remaining varieties were sown between February and April and the varieties Titicaca, Yellow (Amarilla), Black (Negra), Red (Roja) and White (Blanca) were selected. The rest were discarded due to their low germination rate, lax inflorescences or low productivity. So far, the variety Titicaca is the most attractive from an agronomic perspective. The most favorable planting season is at the end of February. During the entire crop cycle, there have been no incidences of pests or diseases and the germination rate of the seeds was high. The Titicaca variety is the only one produced under organic cultivation conditions for its commercialization, and the product is sold in bags of 250 gr in small format, and in cotton bags of 5 kg in large format. Also interesting is the Yellow (Amarilla) variety for its exceptional plant growth at the beginning of its cultivation cycle, exhibiting early flowering and high production, although it is more susceptible to biotic stress. The rest of quinoa varieties tested have not suffered of pests and diseases but high temperatures have reduced the formation of their grain. Among them, the Black (Negra) quinoa is an optimal candidate for pursuing the assays due to its excellent nutritional properties based on results obtained in nutritional trials conducted so far (Doménech and Ros, 2021).

Field trials with amaranth varieties began in 2019. The results obtained to date indicate that Oscar Blanco variety from Peru is the one that has responded best in the assays performed to date, followed by Burganda. The varieties Hipocondriaca and Rojo de Bélgica have also reached an acceptable plant development in all the assays carried out and do not present major problems of pests and diseases.

It is interesting to note that one of the objectives addressed in the project is the elaboration of novel foods with amaranth and quinoa. One of the companies involved in the project (Últimos Panaderos S.L.) has launched two product lines, one with amaranth seeds (variety Kiwicha 1) and the other with quinoa seeds (variety Titicaca). As a result of the tests carried out they are considering to market the amaranth biscuit with chocolate and the vegan meat pie (pastel de carne) with quinoa. In the first case it is a sweet and energetic pastry product, with great palatability. The meat pie is a salty pastry product, which has no lard at the base and top of it, so it is considered suitable for vegans. With this last product, the company is in fact reinventing a typical meat dish from the Region of Murcia for vegans’ consumption.

### Agronomic, physiological and molecular responses to salt stress of amaranth and quinoa

**Figure 5:**
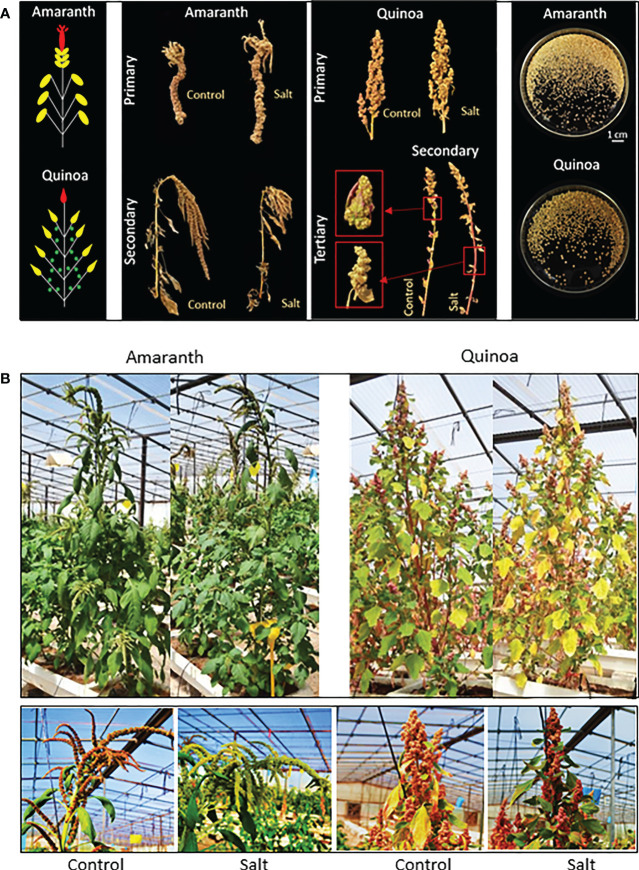
Seed yields are obtained from different panicle types of amaranth and quinoa. **(A)** Left, diagram showing the locations of the main or primary panicle (first panicle to emerge) colored in red, secondary panicles at the tip of each branch (second panicle, emerging after the main panicle) colored in yellow, and for quinoa also tertiary panicles from nodes within branches (third panicle, emerging after the second panicle) colored in green; in center and right, representative images of different panicles and seeds of amaranth and quinoa on the day of harvesting, from plants grown in control and salt stress (100 mM NaCl). **(B)** Phenotype of amaranth and quinoa plants in control condition and after 30 days of salt treatment, and images of main panicles. At this time, the physiological maturity of quinoa plants began to be observed, which does not occur in amaranth. Adapted from Figures 7, 8 of Estrada et al., 2021. Unraveling the strategies used by the underexploited amaranth species to confront salt stress: similarities and differences with quinoa species. Front. Plant Sci. 12:604481. doi: 10.3389/fpls.2021.604481.

In order to advance in the search of new variation sources of tolerance to abiotic stress, we are performing different studies directed to select accessions of quinoa and amaranth able to maintain seed production under multi-stressors conditions. Here we show the results that served as basis to fulfill this objective (Estrada et al., 2021). Within the grain species of amaranth, we selected *A. caudatus* because it has been much less used in studies of salt tolerance than the other two related species (*A. hypocondriacus* and *A. cruentus*). Regarding the stress level, we have selected 100 mM of NaCl for evaluation of the salt tolerance in different assays, as we wish to warrant seed production, a key agronomic trait in both pseudocereal crops, and applying greater stress levels may fatally affect grain production, besides being agronomically unrealistic. Moreover, we have studied the growth responses to salt stress at mid-term on the basis of vegetative growth and at long-term on the basis of seed production with the aim to know whether similar results were obtained to mid- and long-term, and so simplifying the evaluation process for the following materials. At mid-term, assays were carried out in controlled culture conditions and the salt treatment was applied for 20 days, enough time for the plant to show its capacity to recover from both the osmotic and ionic effects of salinity (Campos et al., 2016). In the long-term, the salt tolerance of amaranth and quinoa was evaluated on the basis of grain yield, and assays were carried out during spring-summer in a polyethylene greenhouse with natural conditions located in Santomera municipality (Murcia, Spain), where very high temperatures were achieved (Estrada et al., 2021). The methods applied for obtaining seed yields for each species are showed in the **Figure 5**.

With regard to the issue of how to evaluate the salt tolerance, the results obtained until now do not allow shortening the salt tolerance evaluation to mid-term, as the different degree of salt tolerance between amaranth and quinoa was only observed at long-term. It could be due to the fact that plants suffered of heat stress in our culture conditions to long-term and, therefore, the possibility remains that the results predominantly reflect the heat stress effect instead of that from salt stress, which will be elucidated in future studies. In this regard, Tovar et al. (2020) observed that heat stress affected quinoa seed yield, with yield losses being the result of lower number of seeds produced per plant, as it is observed in our study. On the basis of these results, current studies are being performed to long-term. In spite of the hard work such assays involve, we expect to succeed in selecting elite accessions able to be cultured in harsh stressful conditions, rendering profitable yields with improved quality.

One strategy to simplify the selection of tolerant accessions is to identify the main physiological traits responsible of salt tolerance. Thus, we are analyzing the different anatomical and physiological responses of amaranth and quinoa when confronted to the previously mentioned salt stress treatment (100 mM NaCl) for 20 days, and the expression levels of Na^+^ and K^+^ transporter genes in amaranth, which, up to our knowledge, have not been analyzed until now (Estrada et al., 2021). A key development trait in salt tolerance is the root/shoot ratio (Zou et al., 2022), and precisely the values were much higher in amaranth than in quinoa, up to 50% under salt stress, pointing out that amaranth has a denser root system than quinoa. Although the changes in root/shoot ratio may be genotype-dependent responses, Cai and Gao (2020) observed in quinoa that, rather than developing deep and dense root systems to be able to absorb more water, highland quinoa decreased root growth, therefore avoiding excessive uptake of Na^+^, similarly to the response we have found in our study.

With respect to strategies to maintain the osmotic homeostasis these seem to be different in amaranth and quinoa, as the predominant mechanism in quinoa is its high ability for osmotic adjustment, as it was already observed by Hariadi et al. (2011), while the main mechanism in amaranth seems to be reducing water loss through the leaves. Interestingly, this was accompanied of reduced stomatal density (**Figure 4**). Moreover, we also observed some anatomical changes that may contribute to the reduction of water loss, as increased cell number and reduced cell size, resulting in increasing density of mesophyll cells in leaves (Estrada et al., 2021), which could allow amaranth to augment its capacity to store water. Transpiration efficiency of amaranth was also observed in response to drought stress in accessions of *A. cruentus* (Jamalluddin et al., 2019).

Interestingly, strategies used by amaranth and quinoa to maintain Na^+^ homeostasis under salinity seem to be also divergent, as amaranth exhibited a glycophytic or Na^+^ excluder behavior, contrarily to quinoa, which could be due to the Na^+^ accumulation in EBCs. In fact, Kiani-Pouya et al. (2020) found that different accessions of quinoa exhibiting a low number of EBCs used the strategy of Na^+^ exclusion at the root level, maintaining lower Na^+^ concentration in their leaves upon salinity exposure, a similar strategy to that observed in amaranth. Excluder behavior was also found in other quinoa studies, so a negative correlation between leaf Na^+^ accumulation and plant salinity tolerance was established (Cai and Gao, 2020). Finally, it is interesting the different expression patters of Na^+^ and K^+^ transporters determined in both species, being recently published the first results in amaranth from our laboratory (Estrada et al., 2021). We observed a constitutively remarkable higher expression of all these key genes involved in Na^+^ and K^+^ homeostasis in roots of amaranth compared with quinoa (Estrada et al., 2021), which could be associated to its high availability to regulate Na^+^ homeostasis under salt stress (Katschnig et al., 2015). Regarding *AhHKT1;1*, its highest expression level was found in amaranth root, which could explain the greater retention of Na^+^ in the root of this species compared with quinoa, in spite of the fact that no increased expression was found in saline conditions (Estrada et al., 2021). However, more research efforts are necessary to elucidate the role of the identified amaranth ortholog of *HKT1;1* in salt tolerance of this species.

Currently, we are selecting new materials on the basis of these physiological traits. Moreover, an interesting measure which is simplifying very much the selection process is infrared thermography, a non-destructive analytical tool which has been frequently used in monitoring leaf water loss by transpiration, as previously demonstrated in our study of the salt stress comparative response between the tolerant wild-tomato *S. pennellii* and the salt-sensitive cultivated species *S. lycopersicum* (Albaladejo et al., 2017). These results constitute previous but essential steps for selection of amaranth and quinoa materials to be evaluated for salt tolerance to long-term, on the basis of seed production (Estrada et al., 2021). Finally, all selected materials will be evaluated in the different adverse environmental conditions of the Spanish Southeastern Mediterranean area.

## Concluding remarks

To develop crops with enhanced salt stress tolerance and higher nutritive value, more research toward exploitation of underutilized species is required. Tomato landraces are attractive genetic resources as alternative to commercial cultivars, in order to increase simultaneously stress tolerance and fruit nutritional quality, being necessary to obtain new materials and to delve in studies on the metabolic and molecular responses of tomato fruit to abiotic stress, especially when cultivated in regions where the adverse effects of climate change may be increasingly more intense in short-term, as it is the case of the Mediterranean basin. Moreover, application of modern breeding methods for yield and salt tolerance, such as GWAS, gene editing, and speed breeding, should be considered in order to further advance in this topic. The Spanish Southeast area is the cradle of a set of tomato landraces particularly adapted to these harsh environmental conditions, and we are currently evaluating a collection gathered and registered by the RAERM for stress tolerance and fruit quality. Our results to date demonstrate the huge interest of traditional tomato varieties adapted to the Mediterranean environmental constraints because of their improved fruit quality under salt stress and other abiotic stress growth conditions, which may constitute excellent starting plant materials to develop future biofortified tomatoes adapted to be grown in the current scenario of climate change. Other underexploited species with enormous potential to be used by the agriculture sector to obtain profitable yields under adverse environmental conditions and to improve degraded soils of the Spanish Southeast area are quinoa and amaranth, as the seeds of both pseudocereals have received much attention in recent years because of their exceptional nutritional value. In order to select productive varieties exhibiting high seed quality, the project “Agro-Ecological Innovation Observatory against Climate Change” (AEI-CC) has been performed in the Spanish Southeast region of Murcia, which involves collaborative efforts among scientists and non-academic institutions including those who are actively growing and producing quinoa and amaranth in contrasting environments of this region. The results of this project will serve as a basis for the progress in searching the best varieties, and they are interesting enough with regard to the amaranth grain species *A. caudatus*, which is the species less studied within the grain species. But this is the beginning, as practically no varieties are available by seed companies for their cultivation in the Mediterranean conditions yet. Future implementation of projects of the type of AEI-CC will allow more rapid advances in this direction.

## Author contributions

IE, FF, JE-F, and MB conceptualized the overall structure and wrote the draft manuscript. YE and CF prepared figures and references. IE and YE edited the manuscript. All authors contributed to the article and approved the submitted version.
